# Frailty and Visual Impairment in Elderly Individuals: Improving Outcomes and Modulating Cognitive Decline Through Collaborative Care Between Geriatricians and Ophthalmologists

**DOI:** 10.3390/diseases12110273

**Published:** 2024-11-01

**Authors:** Daniel Dinarvand, Johann Panthakey, Ahmed Hassan, Mohamed H. Ahmed

**Affiliations:** 1 Department of Medicine, Ashford and St. Peter’s Hospital NHS Foundation Trust, Surrey KT16 0PZ, UK; daniel.dinarvand@nhs.net; 2 Department of Medicine, Royal Surrey County Hospital NHS Foundation Trust, Surrey GU2 7XX, UK; johann.panthakey@nhs.net; 3 Faculty of Medicine, Alexandria University, Alexandria 21563, Egypt; ahmed.mohamed2133@alexmed.edu.eg; 4 Department of Medicine and HIV Metabolic Clinic, Milton Keynes University Hospital NHS Foundation Trust, Eaglestone, Milton Keynes MK6 5LD, UK; 5 Department of Geriatric Medicine, Milton Keynes University Hospital NHS Foundation Trust, Eaglestone, Milton Keynes MK6 5LD, UK; 6 Honorary Senior Lecturer of the Faculty of Medicine and Health Sciences, University of Buckingham, Buckinghham MK18 1EG, UK

**Keywords:** frailty, eye disease, old age, dementia, cognitive function

## Abstract

**Introduction**: As life expectancy increases, the prevalence of frailty and eye diseases (cataracts, glaucoma, age-related macular degeneration (AMD), and diabetic retinopathy) in the elderly global population is rising. Eye diseases and visual impairment not only contribute to a high incidence of falls, fractures, depression, and social isolation but they also herald cognitive decline and frailty (vision–cognitive impairment). **Methods**: This narrative review explores the relationship between eye diseases, visual impairment, and frailty, their association with cognitive decline, the current approaches in identifying and managing these conditions and the potential role of interdisciplinary care models. Relevant articles were identified by searching the major databases. **Result**: Eye diseases are common in elderly individuals and can lead to visual impairment and subsequently contribute to falls, fractures, depression, and social isolation. Visual impairment is strongly linked to cognitive decline, which is a key component of frailty. Reduced sensory input from vision loss leads to decreased cognitive stimulation, reduced engagement in activities such as reading, problem-solving, executive function, attention, and social interactions, which are crucial for maintaining cognitive health. This can lead to a form of “sensory deprivation”, which accelerates neurodegenerative processes. As cognitive decline progresses, it creates a feedback loop where individuals may struggle to manage their health, adhere to treatment regimens, or seek timely medical care, exacerbating both cognitive impairment and frailty. Additionally, subjective cognitive decline (SCD) is common in older adults with vision loss and may precede clinical dementia. This sense of declining cognitive ability can worsen anxiety and depression, further contributing to frailty. Early intervention has the potential to mitigate the cognitive effects of vision loss (vision–cognitive impairment). **Conclusions**: Ophthalmologists should play an important role in detecting frailty associated with vision loss. Incorporating frailty assessments into ophthalmic practice can facilitate referrals to geriatric care and early interventions, improving patient outcomes. Geriatricians should be vigilant in identifying visual impairment and referring patients for appropriate ophthalmic investigation and management. Regular vision assessments should be part of comprehensive geriatric evaluations. Future research will assess the beneficial role of community geriatricians in detecting frailty and vision–cognitive impairment. An interdisciplinary and collaborative approach between ophthalmologists and geriatricians can lead to earlier detection, comprehensive management, and improved outcomes in frailty, eye diseases, and cognitive function.

## 1. Introduction

Life expectancy has increased globally in recent decades, reaching an average of over 70 years in 2019, up from 64.2 years in 1990 [1]. As life expectancy increases, there is a corresponding rise in the prevalence of frailty and eye diseases in the elderly population. Frailty affects approximately 18% of community-dwelling adults aged 65 and over, rising to 44% for those aged 85 and over [2]. Cataract is the leading cause of blindness with an estimated 15.2 million cases worldwide. A total of 196 million people are affected by age-related macular degeneration (AMD), and of these, 10.4 million have moderate to severe visual impairment [3]. A total of 76 million people suffer from glaucoma, and this number is expected to rise to 111.8 million by 2040 [4]. With the prevalence of diabetes on the rise, approximately 103 million people have diabetic retinopathy, with 28.4 million having significant visual loss [3]. Uncorrected refractive error is the leading cause of visual impairment worldwide and affects 88.4 million people [3].

Frailty and eye disease negatively affect quality of life and increase the burden placed on our healthcare services, but the two are seldom considered simultaneously or as indicators of each other [5,6]. Eye diseases that are prevalent in elderly individuals include cataract, AMD, glaucoma, diabetic retinopathy and uncorrected refractive error and can all lead to significant visual impairment, contributing to frailty through falls, fractures, depression, and social isolation [6,7]. Frailty is a clinical syndrome that commonly affects elderly individuals and increases their risk of poor health outcomes, including falls, disability, hospitalisation, and mortality. Adverse health outcomes in this population, especially following stressors, are ascribable to the reduced strength, endurance, and physiological function associated with frailty [8,9]. Visual impairment as a consequence of eye disease is one such stressor that can contribute substantially to an increased risk of developing frailty [10,11]. The rise in the prevalence of frailty and eye disease calls for the development of strategies to meet the needs of elderly individuals living with chronic conditions that have the propensity to significantly impact quality of life in order to mitigate the risk of vision–cognitive impairment.

Frailty and visual impairment are associated in various ways. Eye diseases lead to visual impairment and affect balance and coordination, leading to a higher risk of falls, fractures, and hospitalisation and contributing to disability and mortality in elderly individuals [7,10,11,12,13]. Visual impairment can lead to social isolation, depression, and faltering satisfaction with life [14]. It can also reduce an individual’s capacity for social and physical activities, resulting in decreased physical fitness, muscle weakness, and overall deterioration in health [15]. Reduced physical and social engagement, mobility, and cognition associated with visual impairment can accelerate frailty, and therefore, early interventions can mitigate this decline [10,11,13].

Cognitive decline, which is associated with frailty, has been shown to be accelerated by visual impairment (vision–cognitive impairment). Sensory impairments like loss of vision can accelerate cognitive decline and dementia, both of which are associated with frailty [16,17,18]. Traditionally, the literature on frailty has primarily focused on its correlations with physiological capabilities, such as muscle strength and endurance. However, recent research has expanded our understanding of frailty by highlighting its associations with a broader spectrum of factors. These include cognitive performance, unhealthy lifestyles, and poor mental health, along with other psychosocial morbidities like depression and social isolation [19,20,21]. Cognitive performance is now recognised as an important factor in frailty as cognitive decline has been shown to accelerate the progression of frailty. Additionally, cognitive frailty, a condition characterised by the simultaneous presence of both cognitive impairment and physical frailty, has been shown to significantly increase the risk of poor health outcomes such as dementia and all-cause mortality, highlighting the importance of viewing frailty through a multidimensional lens that includes not only physical but also cognitive and psychosocial aspects [19].

Given the economic burden of vision–cognitive impairment, collaboration between ophthalmologists and geriatricians could enhance the provision of comprehensive care for this vulnerable subset of the population affected by both frailty and vision–cognitive impairment. With an interdisciplinary approach, care models that encourage collaboration between these specialities have the potential to lead to earlier detection, holistic management, and improved outcomes for both frailty and vision–cognitive impairment. Integrating tools for the recognition and assessment of frailty and eye disease into routine ophthalmological and geriatric care, respectively, can help clinicians to identify individuals at risk and intervene earlier to mitigate the implications of both visual impairment and frailty, therefore improving health outcomes, reducing healthcare costs, and improving the well-being of elderly patients [22,23,24].

## 2. Methods

**Figure 1:**
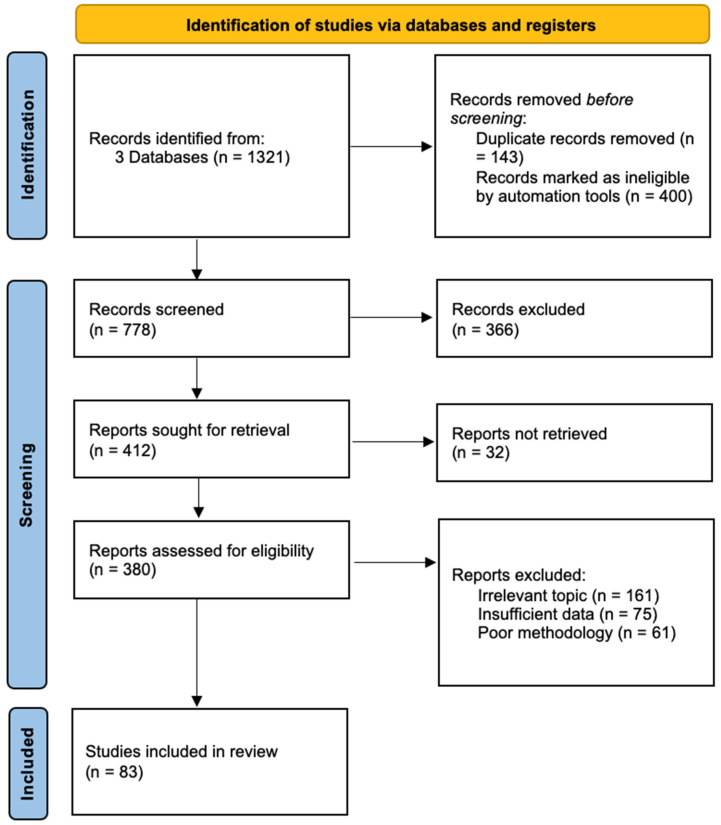
The methodology of this narrative review.

A narrative literature review was conducted to explore the relationship between eye diseases, frailty, and vision–cognitive impairment, as well as the current approaches to identifying and managing these conditions, and the potential role of interdisciplinary care models. Relevant articles were identified through databases such as PubMed, Scopus, and Google Scholar using keywords like “eye disease”, “cataract”, “glaucoma”, “age related macular degeneration”, “diabetic retinopathy”, “frailty”, “geriatric care”, and “interdisciplinary approach”. Selected studies were reviewed to extract data on the prevalence of these conditions, their impact on health outcomes, and examples of integrated care practises (Figure 1).

## 3. Frailty

Frailty is a clinical syndrome defined by a reduction in the physiological reserves of multiple organs and systems in the human body. Individuals with frailty are vulnerable to endogenous and exogenous stressors and have a heightened susceptibility to severe adverse outcomes of acute and chronic diseases acting as stressors on the body. Frailty is therefore a significant barrier to the elderly population living healthier and longer lives and is associated with a higher likelihood of disability, dementia, hospitalisations, institutionalisation, and death [5,8,18,25,26,27,28,29]. Frailty affects approximately 18% of community-dwelling adults aged 65 and over, rising to 44% for those aged 85 and over, with affected individuals facing reduced independence and quality of life [2]. As an individual’s frailty increases, their ability to perform both activities of daily living and instrumental activities of daily living decreases. These include activities such as bathing, dressing, cooking, eating, housekeeping, and managing finances [5]. Individuals who are frail have a higher likelihood and frequency of hospitalisation due to their vulnerability to illness and injury, with each instance causing further physiological decline and the exacerbation of frailty. Hospital admissions in this population are associated with complications like delirium, infections, and immobility and can lead to prolonged recovery, readmission, and permanent functional decline. Patients enter into a cycle of increasing susceptibility to stressors and worsening frailty. Beyond the impact on patients, this can consequently increase the burden on caregivers and healthcare systems, increasing the likelihood of institutionalisation and the need for greater allocation of resources and higher healthcare costs to support affected individuals with rehabilitation and long-term care [26,27,28].

Frailty is associated with cognitive decline and dementia. Frail individuals are at greater risk of developing cognitive disorders, and those with cognitive impairment are more likely to become frail [17,18]. An individual with cognitive decline may be less likely to seek out medical advice and demonstrate reduced concordance with medical plans, therefore complicating the management of their frailty and comorbidities. Therefore, the combination of frailty and cognitive impairment can often lead to worse health outcomes [18]. Individuals who are less able to perform the activities of daily living mentioned previously are at greater risk of requiring institutionalisation in order for their care needs to be met. This further increases their risk of poor health outcomes and diminished quality of life. Frail individuals who are institutionalised often experience an accelerated decline in their health and have increased mortality rates compared with those that are not frail [25,29].

## 4. Visual and Cognitive Impairment and Its Consequences on Elderly Individuals Which Contribute to Frailty

**Figure 2:**
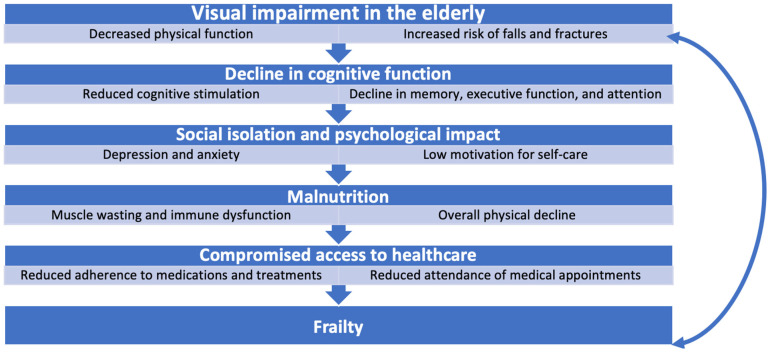
How visual impairment in elderly individuals leads to frailty and the bidirectional relationship between visual impairment and frailty, ultimately leading to vision–cognitive impairment.

Visual impairment is a major public health issue, particularly in ageing populations. The prevalence of visual impairment increases with age and is common in older adults [30]. As life expectancy increases globally, a growing proportion of elderly individuals are living with visual impairments, significantly affecting their quality of life and overall health. The prevalence of visual impairment has risen over time with moderate and severe visual impairment estimated to rise from 217 million in 2015 to 588 million by 2050 worldwide [31]. It is an important healthcare problem and has widespread functional consequences that increase the risk of negative health outcomes [32]. The World Health Organization defines visual impairment as a condition that cannot be fully corrected by refractive solutions such as glasses or contact lenses and often results from conditions such as cataracts, glaucoma, age-related macular degeneration (AMD), diabetic retinopathy, or uncorrected refractive error [3]. Visual impairment has numerous far-reaching impacts on elderly individuals beyond the direct loss of sight. These effects include physical, cognitive, and social dimensions of health, and there is growing evidence linking visual impairment to the development and exacerbation of frailty [33] (Figure 2).

### 4.1. Physical Function, Falls, and Fractures

One of the most pronounced effects of visual impairment in elderly individuals is the deterioration of physical function. Visual impairment limits an individual’s ability to perform essential daily activities, such as dressing, cooking, managing medications, and maintaining personal hygiene [15]. This functional decline often results in increased dependence on caregivers or a transition to institutionalised care, both of which can reduce physical activity and increase sedentary behaviour [6]. Reduced mobility contributes to muscle atrophy, poor coordination, and decreased balance, all of which are risk factors for frailty [10]. As elderly individuals become less active due to their vision-related functional limitations, they enter a cycle of deconditioning that accelerates the onset and progression of frailty [28].

Another important consequence of visual impairment is the heightened risk of falls and fractures. Vision plays a key role in maintaining balance and spatial awareness. Conditions such as cataracts, glaucoma, and age-related macular degeneration can impair depth perception, contrast sensitivity, and peripheral vision, which are essential for navigating one’s environmental safely [12,34,35]. Falls are a major cause of morbidity in older adults, often resulting in fractures that further limit mobility. In particular, hip fractures following falls have devastating consequences, leading to prolonged hospitalisation, loss of independence, and an accelerated decline into frailty [7]. Studies have demonstrated the correlation between visual impairment and an increased risk of falls, reinforcing the physical dangers posed by vision loss in this population [7,34,35,36].

### 4.2. Cognitive Decline

Visual impairment is strongly linked to cognitive decline, which is a key component of frailty [13,37]. Reduced sensory input from vision loss leads to decreased cognitive stimulation, accelerating cognitive deterioration [16]. Vision loss is associated with reduced engagement in activities such as reading, problem-solving, and social interactions, which are crucial for maintaining cognitive health. A meta-analysis by Cao et al. [38] confirmed that visual impairment is associated with poorer outcomes across several cognitive domains, including memory, executive function, and attention. Impaired vision can contribute to cognitive decline by reducing sensory input and limiting engagement in cognitive and social activities. This can lead to a form of “sensory deprivation”, which accelerates neurodegenerative processes. As cognitive decline progresses, it creates a feedback loop where individuals may struggle to manage their health, adhere to treatment regimens, or seek timely medical care, exacerbating both cognitive impairment and frailty [17]. Cognitive impairment and frailty often co-exist, as shown in a study by Li et al. [37], who found that frail individuals exhibited worse cognitive functioning than their non-frail counterparts.

Additionally, subjective cognitive decline (SCD) is common in older adults with vision loss. Subjective cognitive decline describes when older adults may self-perceive a decline in their cognitive abilities relative to a previously normal state. Importantly, this perception occurs despite no observable deficits in age-adjusted, gender-adjusted, or education-adjusted standardised cognitive assessments [39]. SCD is considered a pivotal phase in cognitive preservation and is often viewed as an early indicator of potential cognitive decline, leading to conditions such as mild cognitive impairment (MCI) or dementia [40]. Research suggests that individuals reporting SCD are at higher risk for future cognitive decline, particularly when SCD is accompanied by biomarkers of neurodegenerative diseases, such as amyloid-beta deposition [39]. Addressing SCD in clinical settings is crucial, as early interventions targeting cognitive concerns can help mitigate the progression of more severe cognitive impairments [41].

Luo et al. [42] found that visual impairment increases the risk of SCD, which may precede clinical dementia. This sense of declining cognitive ability can worsen anxiety and depression, contributing further to frailty. A systematic review by Nagarajan et al. [43] emphasised the need for early intervention to mitigate the cognitive effects of vision loss. Overall, research indicates a bidirectional relationship between visual impairment and cognitive decline, highlighting the need for integrated care that addresses both visual and cognitive health. Interventions such as vision rehabilitation may delay cognitive decline, underscoring the importance of comprehensive geriatric assessments in order to mitigate the burden of this new epidemic of vision–cognitive impairment [10,44] (Table 1).

### 4.3. Social Isolation and Psychological Impact

The social and psychological effects of visual impairment further compound its contribution to frailty. Elderly individuals with significant vision loss often withdraw from social interactions due to difficulties in recognising faces or interpreting social cues, which can lead to profound social isolation [14]. Social isolation is a well-documented risk factor for depression, anxiety, and emotional distress, all of which can further exacerbate frailty by decreasing motivation for self-care and physical activity [17]. A study by Rokach et al. [49] highlighted the emotional toll of vision loss, emphasising that loneliness is prevalent among individuals with visual impairment, which negatively impacts their overall well-being. Social isolation not only affects mental health but also leads to reduced engagement in activities that promote physical and cognitive health, thus accelerating the progression of frailty and vision–cognitive impairment.

Huang et al. [50] demonstrated that hearing and vision impairments are significant predictors of social isolation over time, with affected individuals experiencing long-term declines in social interactions and quality of life. This sustained isolation can have a cumulative effect on mental health, particularly in community-dwelling older adults who may already face limited social support. Loneliness and social isolation have been consistently linked to diminished life satisfaction and reduced mental well-being and are significant issues among those with visual impairment. Brunes et al. [51] reported that individuals with visual impairments experience higher levels of loneliness compared to the general population, which is strongly associated with reduced life satisfaction and increased psychological distress.

Depression and anxiety often arise as a consequence of this isolation, further compounding the physical decline associated with frailty. Depression is particularly insidious in its impact on frailty as it can initiate a cycle of physical and emotional decline that is difficult to reverse [14]. These negative psychological outcomes can intensify frailty by reducing motivation for self-care, physical activity, and social engagement, creating a cycle of worsening health [49].

### 4.4. Malnutrition

Malnutrition is another serious consequence of visual impairment in elderly individuals. Vision loss can make it difficult for individuals to shop for groceries, prepare meals, or even eat independently, resulting in inadequate nutrition [10]. Malnutrition is a well-known risk factor for frailty, contributing to muscle wasting, immune dysfunction, and overall physical decline [27]. A recent population-based study by Yang and Lee [52] found that older adults with both visual impairment and diabetes were at a significantly higher risk for poor nutritional status largely due to difficulties in meal preparation and managing dietary needs. These findings underscore the complex relationship between sensory impairments and nutritional risk, which is further exacerbated by chronic health conditions such as diabetes.

Additionally, Cirone et al. [53] highlighted in a systematic review and meta-analysis that dietary insufficiencies, particularly deficiencies in essential nutrients like vitamins A, C, and zinc, are often overlooked as contributing factors to visual impairment. The study emphasised that poor nutrition not only results from vision loss but can also contribute to the progression of certain eye conditions, creating a bidirectional relationship between vision and nutrition. This highlights the importance of dietary interventions for older adults with visual impairment to prevent further deterioration in both vision and overall health. The combination of poor nutrition and reduced physical activity further accelerates the progression of frailty, leaving elderly individuals vulnerable to infection, hospitalisation, and early mortality [52,53].

### 4.5. Compromised Access to Healthcare

Visual impairment can create significant barriers to healthcare access, which, in turn, exacerbates frailty in elderly individuals. People with vision loss may face difficulties managing their medications, attending medical appointments, or following healthcare recommendations. This issue is particularly problematic when compounded by cognitive impairments, as many visually impaired elderly individuals struggle to follow and adhere to complex health regimens [15]. The inability to effectively manage chronic health conditions leads to the worsening of comorbidities, more frequent hospitalisations, and a more rapid decline into frailty. A study by Hou and Pu [54] demonstrated that individuals with visual impairments are more likely to use healthcare services more frequently but often experience delays in receiving necessary interventions such as cataract surgery due to logistical challenges and barriers to accessing timely care. These logistical challenges include the financial burden posed by medical costs associated with visual impairment, particularly for individuals from lower socioeconomic backgrounds who may lack adequate insurance coverage. Patients with visual disabilities also have a higher predicted use of ophthalmic care, increasing wait times and straining healthcare systems. In addition, geographical barriers, particularly in rural areas, exacerbate delays, as patients often need to travel long distances to access care, further delaying necessary interventions such as cataract surgery [54]. These factors contribute to a cycle of health deterioration where untreated conditions exacerbate frailty and increase the risk of adverse outcomes. Visual impairment increases the difficulty of navigating healthcare systems, further complicating the management of chronic diseases. Cupples et al. [55] emphasise the need for tailored interventions to improve healthcare access for individuals with visual impairments, noting that a lack of accessible health information, such as medication instructions in large print or audio formats, impedes the ability of patients to adhere to their treatment regimens. This lack of accessible healthcare resources contributes to poorer health outcomes and accelerates frailty and vision–cognitive impairment (Figure 2). While audio formats and large print are valuable, other accessible formats like Braille can significantly enhance cognitive development in visually impaired individuals, as reading and writing in Braille engages different cognitive pathways compared to listening alone [55,56]. This underscores the need for tailored interventions that not only provide accessible health information but also promote cognitive development through diverse formats.

The challenges faced by visually impaired individuals extend beyond physical barriers to healthcare settings. Timilsina et al. [57] explore the healthcare access challenges faced by visually impaired women, identifying issues such as stigma, sexual harassment, and inadequate support within healthcare environments. These challenges, while contextual, highlight the global difficulties that people with visual impairments encounter when seeking medical care. The additional psychological and emotional burden of navigating such environments can discourage individuals from accessing care, thereby worsening their health status and contributing to frailty. Poor adherence to medical regimens, compounded by delays in receiving care and the complexities of navigating healthcare systems, leads to a cycle of health deterioration. Without timely interventions, this deterioration accelerates frailty, leaving elderly individuals more vulnerable to worsening health conditions and a decreased quality of life [10].

## 5. The Bidirectional Relationship Between Vision–Cognitive Impairment and Frailty

The relationship between vision–cognitive impairment and frailty is intertwined, with each condition amplifying the effects of the other. Vision loss increases vulnerability to frailty by limiting physical activity, accelerating cognitive decline, contributing to social isolation and malnutrition, and complicating healthcare management. A systematic review and meta-analysis by Ripa et al. [45] confirmed that visual impairment is a significant risk factor for the development of frailty in older adults, as vision loss exacerbates physical and functional decline. Similarly, Shang et al. [58] demonstrated in a longitudinal study that older adults with visual impairment were more likely to develop frailty, with the relationship being particularly pronounced among individuals with severe visual impairment.

Research has also shown that frailty worsens the challenges associated with vision–cognitive impairment. Frail individuals are often less likely to undergo necessary interventions, such as cataract surgery, which can lead to further visual decline [15]. Frailty is associated with decreased access to surgical care, influenced by factors such as health system barriers, lower healthcare utilisation, and physical limitations, and it should be noted that disparities in healthcare access further delay necessary procedures. Frail individuals often face barriers to healthcare services due to cognitive and physical limitations, which contribute to delayed or missed surgeries [59,60]. Frail patients often experience delays in cataract surgery due to health system inefficiencies and the under-utilisation of care, exacerbating visual impairment and frailty [61]. In some cases, frailty complicates postoperative recovery, reducing the efficacy of treatments intended to restore or improve vision. Age-related factors and comorbidities increase the complexity of recovery after surgical interventions. Elderly individuals, especially those with frailty, face additional challenges in recovery due to the compounded effects of ageing and multiple comorbidities. This underscores the need for tailored, postoperative care that takes into account the physiological changes in frail individuals. Enhanced recovery protocols specifically designed for the geriatric population can improve outcomes and reduce complications, particularly in frail patients undergoing surgeries [62]. The feedback loop between frailty and visual impairment results in progressively worsening health outcomes and diminished quality of life for elderly individuals, as emphasised by Liljas et al. [11]. In a study of community-dwelling older adults, Hou et al. [22] found a bidirectional association between visual impairment and frailty, noting that frail individuals were also more likely to develop vision loss over time (Figure 2). This relationship indicates that frailty not only contributes to the progression of visual impairment but also arises as a consequence of untreated or inadequately managed vision problems. Liljas et al. [63] similarly observed that self-reported vision impairment was a predictor of both incident prefrailty and frailty in older adults over a four-year period, reinforcing the need for early intervention.

The cumulative impact of these two conditions is substantial as the combination of frailty and vision–cognitive impairment leads to greater dependency, poorer health outcomes, and higher healthcare costs. Given this close association, integrated care approaches are essential for mitigating the impact of both conditions. The early identification of at-risk individuals is crucial, as interventions targeting either vision impairment or frailty can potentially slow the progression of both conditions. For instance, Shang et al. [58] demonstrated that addressing vision impairment could lead to improved physical function, which, in turn, reduces the risk of frailty.

Given the close association between vision–cognitive impairment and frailty, early intervention and interdisciplinary care are essential for improving outcomes (Table 2). Collaboration between ophthalmologists, geriatricians, and other healthcare professionals, including physiotherapists, occupational therapists, psychologists, and nutritionists, can facilitate the earlier detection and management of both conditions, ensuring that elderly individuals receive comprehensive care. Integrating frailty assessments into routine ophthalmological care and vision assessments in geriatric care may help break the cycle of vision–cognitive impairment and frailty, ultimately improving quality of life and reducing healthcare costs [25]. This proactive approach has the potential to improve the quality of life and reduce the healthcare burden associated with these dual conditions [45].

## 6. Common Eye Diseases Affecting Elderly Individuals

### 6.1. Cataracts

Cataract is the opacification of the crystalline lens that leads to blurred vision, glare, sensitivity to lights, and difficulty seeing in low light conditions. Cataracts are primarily caused by the normal ageing process but can also result from trauma, metabolic disorders, medications, or congenital problems [64]. Cataracts are the leading cause of blindness worldwide, affecting over 65 million people, with the majority of cases occurring in individuals over the age of 60. The prevalence of cataracts increases dramatically with age, and by the age of 80, over 50% of individuals in high-income countries have cataracts or have undergone cataract surgery [3]. In elderly individuals, cataracts are particularly concerning because they contribute to reduced physical activity and an increased risk of falls due to impaired vision [65]. The primary treatment for cataracts is surgical removal of the opacified crystalline lens, which is replaced with an artificial intraocular lens (IOL). Cataract surgery is highly effective and generally very safe [64].

### 6.2. Age-Related Macular Degeneration

Age related macular degeneration (AMD) is a potentially progressive maculopathy characterised by distinct clinical stages, including early and intermediate AMD with drusen and macular pigmentary changes and late AMD, which is associated with a decrease in or loss of central vision. Late AMD can be classified into geographic atrophy (dry AMD) and neovascular AMD (wet AMD). Dry AMD is characterised by the accumulation of drusen in the retina, leading to gradual vision loss, while wet AMD involves choroidal neovascularisation, often causing more rapid and severe visual impairment due to bleeding or the leakage of fluid [66]. AMD is the leading cause of irreversible vision loss in individuals over the age of 60. Globally, around 196 million people were affected by AMD in 2020, with this number being expected to rise as the population ages [3]. The risk of developing AMD increases with age, and frail elderly individuals are particularly vulnerable due to comorbidities such as cardiovascular disease, which is linked to the progression of AMD [31].

There is no cure for dry AMD, but the progression of the disease can be slowed with the use of high-dose antioxidant vitamins and minerals, as recommended by the Age-Related Eye Disease Study (AREDS). Wet AMD is treated with intravitreal anti-VEGF (vascular endothelial growth factor) injections, which inhibit abnormal blood vessel growth in the retina. Regular monitoring and early intervention are key to managing AMD, although frail patients may face challenges in attending frequent medical appointments for injections. For advanced cases, low-vision aids such as magnifiers or specialised lighting can help improve functional vision [66].

### 6.3. Primary Open-Angle Glaucoma

Glaucoma is a neurodegenerative condition primarily due to dysfunction in the outflow of aqueous humour, the nutrient-rich fluid that constantly flows through the eye, resulting in an increase in intra-ocular pressure, and it is characterised by retinal ganglion cell damage, peripheral vision loss in early disease, and central vision loss in late disease [67]. Globally, approximately 76 million people are affected by glaucoma, and this number is expected to increase to 111.8 million by 2040 [4]. The risk of glaucoma increases with age, particularly in individuals over the age of 60. Frail elderly individuals are at a higher risk of undiagnosed glaucoma due to reduced access to routine eye care and difficulties with early symptom recognition, as the disease is often asymptomatic in its early stages [13].

Glaucoma is typically managed through the use of medications, such as topical eye drops to lower intraocular pressure (IOP), or surgical interventions like trabeculectomy or laser procedures. The goal of treatment is to prevent further optic nerve damage and preserve remaining vision. In frail patients, adherence to treatment regimens may be challenging due to cognitive or physical impairments, and surgical risks are higher. Regular monitoring of IOP and visual fields is essential to managing the disease, though frailty may limit the ability of elderly individuals to attend regular follow-up appointments [67].

### 6.4. Diabetic Retinopathy

Diabetic retinopathy is the chronic progressive retinal manifestation of hyperglycaemic vascular damage and neurodegenerative change. Diabetic retinopathy progresses through four stages, from mild non-proliferative changes to proliferative retinopathy, which involves abnormal blood vessel growth that can lead to severe vision loss or blindness [68]. Diabetic retinopathy affects approximately 103 million people globally, with 28.4 million experiencing significant vision impairment [3]. As diabetes becomes more prevalent, especially among ageing populations, the burden of diabetic retinopathy is expected to increase. Frail individuals with diabetes are at a heightened risk of developing more severe forms of retinopathy due to poorer glycaemic control and other comorbidities that complicate disease management [69]. 

The management of diabetic retinopathy involves optimising blood glucose levels, blood pressure, and cholesterol to prevent further microvascular damage. In more advanced cases, treatments such as laser photocoagulation, intravitreal anti-VEGF injections, or vitrectomy may be required [68]. For frail individuals, maintaining proper glycaemic control can be challenging, and regular eye examinations may be neglected due to mobility issues or cognitive decline. Coordinated care between endocrinologists, ophthalmologists, and geriatricians is essential for managing diabetic retinopathy in frail populations [10].

### 6.5. Uncorrected Refractive Error

Uncorrected refractive error, which includes conditions such as myopia, hypermetropia, and astigmatism, is the leading cause of visual impairment globally. These errors occur when the eye does not focus light onto the retina, resulting in blurred vision. While refractive errors can be easily corrected with glasses, contact lenses, or refractive surgery, many elderly individuals do not receive the necessary care to address these conditions. Refractive errors affect over 88 million people globally [3]. In elderly populations, the prevalence of uncorrected refractive errors is high, particularly in low-resource settings where access to spectacles or eye care services is limited. The management of refractive errors in elderly individuals typically involves prescription spectacles or contact lenses. For frail patients, regular eye examinations are crucial to ensuring that their refractive needs are met. However, barriers to accessing eye care services, such as transportation difficulties or financial constraints, may prevent frail individuals from receiving appropriate corrective lenses [10].

## 7. Vision–Cognitive Impairment and Frailty in Developing Countries

The burden of visual impairment and frailty in developing countries is substantial and presents unique challenges due to limited healthcare resources, socioeconomic constraints, and underdeveloped healthcare infrastructure. In these settings, elderly populations are often underserved, with restricted access to specialised eye care and geriatric services. This exacerbates the impact of both visual impairment and frailty on individuals and communities. Visual impairment is highly prevalent in developing countries due to common conditions such as cataracts, uncorrected refractive errors, glaucoma, and diabetic retinopathy [3,31]. A study by Abraham et al. [70] found that individuals with HIV in ageing populations are at an increased risk of visual impairment, further compounding their healthcare needs in resource-limited settings. The lack of timely and affordable eye care means many elderly individuals live with untreated visual impairment, leading to preventable blindness, reduced quality of life, increased dependency on others, and, ultimately, rapid cognitive impairment.

Frailty is also prevalent in older adults in developing countries, driven by factors such as malnutrition, chronic disease, limited healthcare access, and a lack of social support systems [26]. Visual impairment and frailty have a cyclical relationship, where vision loss increases the risk of frailty due to reduced physical activity, malnutrition, social isolation, and falls [10]. For example, Singh and Maurya [47] identified that visual impairment is a significant predictor of falls in older adults in India, which contributes to increased morbidity and accelerated frailty and cognitive impairment. In Central Africa, frailty and visual impairment are closely linked, particularly among older individuals with cognitive decline. The EPIDEMCA study [46] highlighted that self-reported vision impairment was strongly associated with frailty in older adults who exhibited low cognitive performance. This demonstrates the vulnerability of ageing populations in resource-limited settings, where untreated vision issues exacerbate both frailty and cognitive decline.

The primary barriers to addressing visual impairment and frailty in developing countries include limited healthcare infrastructure, insufficient trained professionals, and high costs of care. Rural and remote areas are often underserved by healthcare services, meaning many elderly individuals do not receive necessary interventions such as cataract surgery, prescription eyeglasses, or treatment for age-related macular degeneration [69]. This results in a significant number of untreated vision impairments that lead to blindness and increase the risk of frailty.

Economic barriers also play a key role. In many low-income countries, healthcare services, including eye care, are unaffordable or inaccessible for older adults, particularly those with limited mobility. This lack of access to preventive care contributes to the progression of both frailty and visual impairment, trapping individuals in a cycle of health decline [54].

Community-based interventions offer a promising strategy to mitigate the impact of visual impairment and frailty in developing countries. Programmes that bring vision screening and eye care services to rural areas via mobile clinics or telemedicine have improved access for underserved populations [11]. These interventions provide timely diagnosis and treatment for conditions like cataracts and uncorrected refractive errors while also educating communities on the importance of eye health and the early detection of frailty. In regions with limited healthcare professionals, training non-specialist healthcare workers to conduct basic vision screenings and frailty assessments can significantly expand access to care. This approach can help detect early signs of vision loss and frailty, enabling referrals to ophthalmologists and geriatricians when needed [63].

Emerging technologies, such as smartphone-based vision screening apps and teleophthalmology, hold the potential to revolutionise eye care delivery in developing countries. These tools enable healthcare providers to remotely diagnose and monitor visual impairment, reducing the need for patients to travel long distances for care. In addition, telemedicine platforms facilitate interdisciplinary collaboration between ophthalmologists, geriatricians, and other healthcare professionals, ensuring that frail elderly patients with visual impairment receive comprehensive care [57]. Additionally, affordable interventions, such as low-cost spectacles and cataract surgery programmes, can greatly improve visual outcomes for elderly individuals in these regions. International initiatives, such as VISION 2020, have made significant progress in reducing the burden of blindness in low-resource settings. However, more work is needed to integrate frailty screening and management into these programmes [3].

Malnutrition is a significant contributor to frailty and is often exacerbated by visual impairment. Older adults with vision loss face challenges in shopping for food, preparing meals, and eating independently, which leads to poor nutrition and subsequent frailty [53]. Poor nutrition is also associated with the progression of age-related eye diseases, such as cataracts and macular degeneration. Socioeconomic factors, including poverty and limited access to social support, further exacerbate the cycle of frailty and vision–cognitive impairment. Policies aimed at improving social security for older adults and providing affordable healthcare are crucial for reducing this burden in developing countries. In developing countries, the intersection of vision–cognitive impairment and frailty presents a significant public health challenge that requires targeted interdisciplinary interventions. Expanding access to eye care services, utilising community health workers, leveraging technology, and addressing socioeconomic and nutritional factors are essential steps in mitigating the impact of these conditions.

## 8. The Role of Screening for Frailty in Ophthalmology

Screening for frailty can help to identify patients who are at risk of poor surgical outcomes, delayed recovery, or complications related to age-associated decline [71]. While ophthalmic surgeries are typically considered low-risk, frailty increases susceptibility to adverse outcomes, even in relatively low-risk procedures such as cataract surgery [72]. The early identification of frailty can therefore enable ophthalmologists to make informed decisions regarding surgical risks and postoperative care. Vulnerable individuals can be referred to geriatric services for further comprehensive assessment and management [73]. Whilst many frailty assessment tools exist, the Clinical Frailty Scale (CFS) offers a practical and efficient way to identify frail or prefrail patients outside of the geriatric care setting.

### Clinical Frailty Scale (CFS)

The Clinical Frailty Scale (CFS) is a simple and widely used frailty assessment tool that grades a patient’s level of frailty on a scale from 1 (very fit) to 9 (terminally ill) based on the individual’s overall health status [74,75]. The CFS is designed to provide a quick, intuitive assessment of frailty, making it well suited for clinical settings where time and resources are limited [75,76]. It has been validated in several studies for its use in the care of elderly individuals, including in the context of surgical decision making [75,77]. The CFS can be particularly valuable in ophthalmology because it offers a holistic assessment of a patient’s overall health along with parameters including physical, cognitive, and social function, all of which are relevant to frailty [75]. Vision loss in elderly patients can exacerbate cognitive decline and affect physical functioning, increasing the importance of screening for frailty in this subset of patients. In preoperative settings, the CFS can help to identify frail patients who may be at a higher risk of complications and guide decisions on the timing and approach to surgery [78]. For instance, an elderly patient presenting for cataract surgery with a moderate CFS score may require additional perioperative planning, such as closer postoperative monitoring or referral to geriatric services for nutritional or mobility support. Frail patients often require multidisciplinary care to manage their health effectively, including interventions aimed at improving functional status and reducing polypharmacy [5,75].

The primary strength of the CFS in this setting is its simplicity and speed, making it a practical tool for use in a busy ophthalmology clinic [77]. Its holistic nature allows clinicians to account for cognitive and functional impairments, which are crucial considerations in elderly patients with visual impairment. The subjective nature of the CFS can introduce some variability in assessment depending on the clinician’s judgement or the patient’s presentation [74]. Despite this, studies have demonstrated its utility with intraclass correlation coefficients (ICC) above 80% [79,80] and Cohen’s kappa values ranging between 0.74 and 0.85 [81]. It therefore remains a valuable screening tool for identifying patients who require further geriatric evaluation.

Implementing routine frailty screening with the use of a tool like the CFS in ophthalmology settings has the potential to improve patient outcomes by identifying those at increased risk for complications related to eye surgeries. The early identification of frailty allows for timely referrals to geriatric services, where comprehensive assessments can guide interventions that reduce surgical risks, improve postoperative recovery, and optimise overall patient care [5]. Integrating frailty screening into routine ophthalmological care should be considered the best practice for ensuring that frail individuals receive the holistic care they need to maintain their quality of life and functional independence.

## 9. The Role of Screening for Visual Impairment in Elderly Individuals by Geriatricians

Visual impairment is a common and significant health issue in the elderly population, often leading to an increased risk of frailty, reduced quality of life, and greater dependence on healthcare services [10]. The early identification and treatment of visual impairment in this population can play an important role in preventing the progression of frailty and improving overall well-being [10,63]. Given the close relationship between frailty and visual impairment, screening for visual impairment by community geriatricians or hospital-based geriatricians can represent a key intervention in the continuum of elderly care. As individuals age, they become more susceptible to common vision problems such as cataracts, age-related macular degeneration (AMD), glaucoma, and diabetic retinopathy [3]. If left untreated, these conditions not only lead to deteriorating vision but also contribute to increased physical inactivity, social isolation, depression, and ultimately frailty [17]. Early screening for visual impairment is particularly important because many elderly individuals may not recognise the gradual loss of vision as a significant issue until it substantially impairs their daily activities [45].

Geriatricians, whether working in community settings or hospital environments, are at the frontline to screen for visual impairment during routine assessments of elderly individuals and then refer them to ophthalmologists for ongoing management. We propose that vision screening can be easily integrated into broader health assessments, such as during comprehensive geriatric evaluations, where other domains like mobility, cognition, and nutrition are already being examined [82,83]. Identifying visual impairment in this context allows geriatricians to refer patients promptly to ophthalmology services, where they can receive appropriate treatment for their eye disease. Geriatricians can ask patients if they have difficulties with vision-related activities, such as reading, driving, or recognising faces, which may indicate the need for further ophthalmologic evaluation. Visual impairment screening by geriatricians can include simple tests such as visual acuity checks, contrast sensitivity testing, and basic assessments for common conditions like cataracts, AMD, or glaucoma.

**Figure 3:**
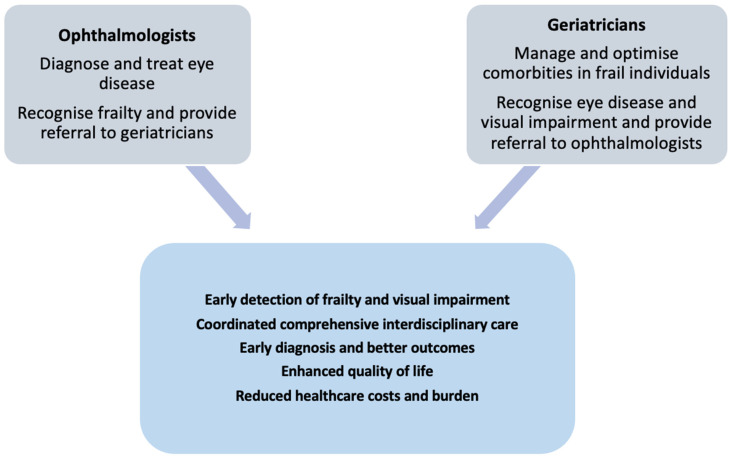
The role of ophthalmologists and geriatricians in improving patient outcomes through an interdisciplinary approach.

Vision loss restricts physical activity by impairing mobility and increasing the risk of falls, which, in turn, leads to muscle atrophy, physical decline, and an increased likelihood of becoming frail [12]. By screening for and addressing visual impairment, geriatricians can help reduce these risks. Early intervention, such as cataract surgery or treatments for AMD and glaucoma, has been shown to improve physical function and reduce the risk of developing or worsening frailty [7]. Restoring vision improves an individual’s ability to perform activities of daily living, including reading, cooking, and navigating their environment, providing greater independence and reducing the burden on caregivers [10]. Improved vision also enhances social interactions by enabling individuals to recognise faces and engage more fully in social activities, reducing the risk of isolation and depression [51]. These benefits directly counter some of the key psychosocial contributors to frailty. Screening for and treating conditions like cataract, which affects contrast sensitivity and depth perception, can significantly reduce the risk of falls and related injuries. Studies have demonstrated that cataract surgery, for example, not only improves vision but also decreases fall risk, providing a dual benefit to physical health and safety [48]. Geriatricians are already accustomed to assessing and managing chronic diseases, cognitive decline, mobility issues, and frailty in an integrated manner [73]. Adding basic visual screening to this list enhances the scope of care and enables geriatricians to provide a more comprehensive approach to managing the health of older adults. This holistic approach, combined with a timely referral to ophthalmology services, ensures that vision is not overlooked in the care of older adults, thereby promoting better outcomes across various health domains (Figure 3).

## 10. What Is the Proposed New Role for Community Geriatricians in the Campaign Against Vision–Cognitive Impairment?

Community geriatricians represent a vital part of the workforce in the geriatric specialty, focusing on care for elderly individuals within the community. We propose expanding their role to include the coordination and facilitation of screening for frailty and vision–cognitive impairment in collaboration with other healthcare professionals rather than assuming the responsibility of visual screening themselves. Community geriatricians can work closely with opticians, community nurses, and general practitioners to ensure comprehensive care. Geriatricians can utilise the expertise and experience of opticians by working collaboratively to identify at-risk individuals. For example, most opticians maintain records of their older patients, including when they are due for eye assessments. Additionally, some opticians visit elderly patients at home to conduct eye tests. Community geriatricians may partner with opticians in developing initiatives, such as “Towards Improved Visual & Cognitive Health”, which aims to improve outcomes for elderly patients by integrating frailty and vision–cognitive screening efforts into broader geriatric care (Figure 3).

## 11. Future Directions

There is a growing need for ophthalmologists who specialise in visual impairment in the elderly, particularly as this population continues to expand. With life expectancy increasing, it is essential that future ophthalmology training programmes incorporate geriatric ophthalmology into their curricula, mirroring the need for geriatricians to be well versed in screening for visual impairment. Ophthalmologists should be able to carefully consider how frailty may affect the outcomes of their patients who are undergoing treatment for visual impairment. The same also apply for opticians. This interdisciplinary approach can enhance patient care by addressing the overlap between visual decline and frailty. Therefore, it is also important to expand in training other healthcare professionals in basic vision assessment like occupational therapists and physiotherapists.

**Figure 4:**
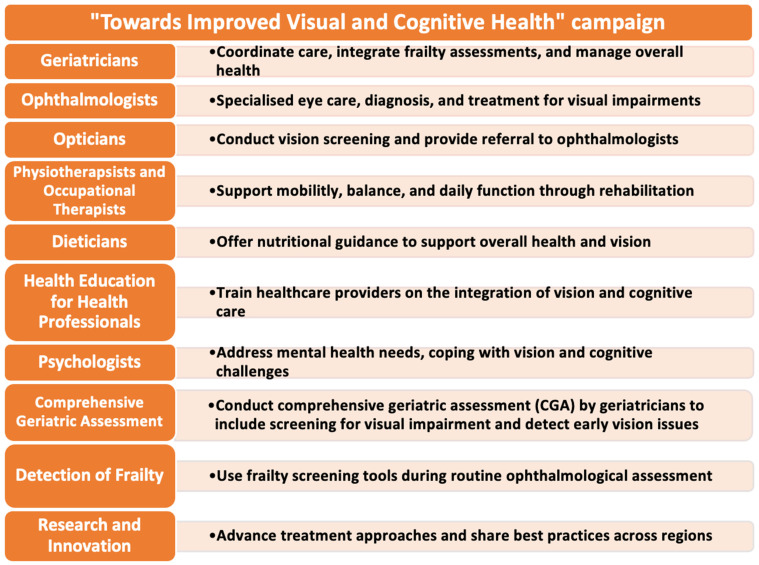
Future directions in the management of vision–cognitive impairment. There is an urgent need to modify the roles of healthcare professionals to include assessments of frailty and vision. A comprehensive geriatric assessment (CGA) can be utilised to assess vision. Health education and collaboration among health professionals will lead to the success of the campaign “Towards Improved Visual and Cognitive Health”.

Heath education and increasing awareness among the public and healthcare professionals of the value of the campaign “Towards Improved Visual & Cognitive Health” are important (Figure 4). Dieticians and psychologists may play crucial roles in mitigating the impact of visual decline. Their roles can be extended to teach relatives about the best strategies to help in minimising cognitive decline. In addition to curriculum advancements, large-scale interventional studies are needed to assess the role of emerging technologies, such as mobile devices, in stimulating cognitive and brain function in elderly individuals with visual impairment. Mobile health applications, virtual reality, and brain stimulation technologies are promising tools for maintaining cognitive engagement and improving outcomes for this population. Further research should also explore the potential of other innovative interventions, such as telemedicine, wearable devices, and AI-driven diagnostic tools, to enhance vision care in older adults (Figure 4).

At the cellular level, additional studies are required to investigate how visual impairment affects neurotransmitter function and neural plasticity in the ageing brain. Understanding these mechanisms may reveal new therapeutic targets for both visual and cognitive decline, offering a more comprehensive approach to managing frailty. Finally, identifying the best strategies for rehabilitation in this population is crucial. This includes designing tailored rehabilitation programmes that address both visual and physical impairments, optimising patient independence, and enhancing quality of life. Research into multidisciplinary rehabilitation models, combining physical therapy, cognitive training, and vision rehabilitation, will be important in shaping future care for elderly individuals at risk of both frailty and visual decline. This will necessitate the need for collaboration between academic centres not only in developing countries but also in developed countries (Figure 4).

## 12. Conclusions

The increasing global life expectancy, along with the rising prevalence of chronic conditions such as frailty and visual impairment in the elderly population, demands an integrated approach to care. This review has highlighted the profound and bidirectional relationship between vision–cognitive impairment and frailty, emphasising how untreated visual disorders can accelerate physical decline, cognitive deterioration, and social isolation, thereby exacerbating frailty. The early detection and treatment of visual impairment by geriatricians through routine screenings can lead to timely referrals to ophthalmology services, where appropriate interventions can be administered. Screening for frailty in ophthalmology settings can help identify vulnerable patients who may require additional support, both during and after surgery, to prevent further decline. Vision assessments should be part of the comprehensive geriatric evaluation. We hope this review will inspire community geriatricians to lead the campaign “Towards Improved Visual & Cognitive Health”.

## Figures and Tables

**Table 1:** Summary of studies linking vision impairment with cognitive impairment.

Authors/Year/Reference	Sample Features	Assessments/Tools	Main Outcomes
Ripa et al., 2022 [45]	Included 12 studies on elderly populations aged 65+	Vision impairment assessments, frailty scales	This systematic review and meta-analysis examined the bidirectional relationship between visual impairment and frailty, emphasising that visual impairment leads to physical and cognitive decline and social isolation, which contribute to frailty. Frailty exacerbates visual impairment due to limited access to care, creating a feedback loop. Frailty progression was linked to worsened health outcomes, including higher mortality rates.
Cao et al., 2023 [38]	Older adults aged 65+, multiple regional data	Cognitive assessments, vision impairment tests	This meta-analysis linked vision impairment with cognitive decline, highlighting associations across several cognitive domains (memory, executive function, and attention). Vision rehabilitation was noted as an intervention to delay cognitive decline and reduce frailty risk.
Lin et al., 2004 [16]	1500 elderly women (aged 65+) from USA	Vision and hearing impairment assessments, cognitive performance tests	This prospective cohort study examined sensory impairments, including vision loss, as predictors of cognitive and functional decline in older adults. Combined vision and hearing impairments increased the likelihood of frailty, reinforcing the importance of integrated care to address both sensory impairments and frailty.
Nagarajan et al., 2022 [43]	Elderly populations aged 65+, global scope	Vision impairment assessments, frailty scales	This systematic review linked vision impairment with cognitive impairment, finding that vision impairment increases the risk of cognitive decline and frailty. Vision rehabilitation could improve functional capacity and delay cognitive decline, highlighting the importance of early intervention.
Swenor et al., 2020 [10]	Elderly participants aged 65+, USA	Vision impairment tests, frailty scales	This longitudinal study found a significant association between visual impairment and frailty. Older adults with visual impairment were more likely to develop frailty, driven by decreased physical activity, cognitive decline, and social isolation. Addressing vision impairment could slow frailty progression.
Varadaraj et al., 2021 [13]	Elderly participants aged 60–85, USA	Vision impairment tests, cognitive function assessments	This longitudinal study explored the association between vision impairment and cognitive decline, showing that visual impairment was linked to poorer performance across cognitive domains (memory and executive function). The authors recommended interventions like cataract surgery to improve both vision and cognitive function, potentially mitigating frailty.
Gbessemehlan et al., 2021 [46]	Elderly individuals with low cognitive performance, Central Africa	Self-reported vision impairment, frailty scales	This cross-sectional study explored the association between vision impairment and cognitive decline, showing that visual impairment was linked to poorer performance across cognitive domains (memory and executive function). The authors recommended interventions like cataract surgery to improve both vision and cognitive function, potentially mitigating frailty.
Liljas et al., 2016 [11]	Older adults aged 60+ in UK	Vision impairment assessments, physical performance tests	This longitudinal study in older adults found that visual impairment significantly predicted both prefrailty and frailty. Early intervention through vision screening and management was key to preventing frailty and improving health outcomes.
Singh et al., 2022 [47]	Longitudinal study	Vision impairment tests, fall risk assessments	This longitudinal study conducted in India demonstrated that visual impairment significantly increased the risk of falls in older adults, which, along with frailty, increased fall risk. Falls and fractures further accelerated frailty. This study recommended interventions to reduce falls and mitigate frailty in older adults with visual impairment.
Gutiérrez-Robledo et al., 2021 [48]	Elderly individuals undergoing cataract surgery	Fall risk assessments, vision function assessments	This systematic review and meta-analysis focused on the impact of cataract surgery on falls among older adults. Cataract surgery significantly reduced the risk of falls and, consequently, frailty progression. Restoring vision via surgery was noted as an effective intervention to improve physical function and mitigate frailty risk.
Hou et al., 2022 [22]	Community-dwelling older adults	Vision impairment assessments, frailty scales	This longitudinal study examined the bidirectional relationship between visual impairment and frailty. It highlighted that individuals with visual impairment are at a greater risk of developing frailty, and frail individuals are more likely to suffer from cognitive decline, forming a feedback loop between the two conditions.

**Table 2:** The bidirectional relationship between frailty and common eye diseases in elderly individuals.

Eye Condition	Explanation of Bidirectional Relationship Between Eye Condition and Frailty	References
Cataracts	Physical limitations caused by cataracts can lead to muscle atrophy and the loss of independence, which are key drivers of frailty. Frail individuals are less likely to undergo cataract surgery due to concerns about surgical risks and recovery. Delaying surgery further exacerbates both vision–cognitive impairment and frailty. Additionally, studies have shown that cataract surgery can reduce the risk of falls and improve quality of life.	[10,48,64,65]
Age-Related Macular Degeneration	AMD primarily affects central vision, making activities such as reading, recognising faces, and driving difficult. This loss of independence can lead to social isolation, depression, and reduced physical activity, contributing to frailty associated with vision–cognitive impairment. Frailty complicates AMD management as frail individuals with vision–cognitive impairment may struggle to attend regular appointments for anti-VEGF injections or adhere to prescribed treatments. Furthermore, frail individuals often have cardiovascular comorbidities, which can exacerbate AMD progression.	[10,31,66]
Primary open-angle glaucoma	Glaucoma causes progressive peripheral vision loss, leading to challenges in mobility, an increased risk of falls, and reduced independence, all of which contribute to frailty. Frailty can make it difficult for patients to adhere to glaucoma treatments such as eye drops, and frail individuals often face challenges attending regular monitoring appointments. Additionally, untreated glaucoma accelerates visual and functional decline, exacerbating cognitive impairment and frailty.	[4,10,67]
Diabetic retinopathy	Diabetic retinopathy is associated with visual impairment, which makes it difficult for individuals to perform daily activities, exacerbating frailty. Frail individuals often struggle with proper diabetes management, leading to worse glycaemic control, which accelerates the progression of retinopathy. This creates a cycle where vision deteriorates, physical activity decreases, and the risk of frailty and associated vision–cognitive impairment increases. Frailty also reduces the ability to attend regular eye checkups for timely interventions.	[10,31,68,69]
Uncorrected refractive error	Uncorrected refractive errors, such as myopia or hyperopia, impair daily activities like reading, cooking, and walking safely. This limitation increases the risk of social isolation, depression, and physical inactivity, driving the progression of frailty. Frail individuals often face barriers to accessing eye care services, which can prolong uncorrected vision and worsen functional decline, further accelerating frailty and vision–cognitive impairment. The early correction of refractive errors can improve independence and reduce frailty risk.	[3,10,31]

## Data Availability

All available information is included in this review.
